# Functional approach to high-throughput plant growth analysis

**DOI:** 10.1186/1752-0509-7-S6-S17

**Published:** 2013-12-13

**Authors:** Oliver L Tessmer, Yuhua Jiao, Jeffrey A Cruz, David M Kramer, Jin Chen

**Affiliations:** 1 Department of Computer Science and Engineering, Michigan State University, East Lansing, Ml 48864 USA; 2 Department of Biochemistry and Molecular Biology, Michigan State University, East Lansing, Ml 48864 USA; 3 MSU-DOE Plant Research Laboratory, Michigan State University, East Lansing, Ml 48864 USA

## Abstract

**Method:**

Taking advantage of the current rapid development in imaging systems and computer vision algorithms, we present HPGA, a **h**igh-throughput **p**henotyping platform for plant **g**rowth modeling and functional **a**nalysis, which produces better understanding of energy distribution in regards of the balance between growth and defense. HPGA has two components, PAE (Plant Area Estimation) and GMA (Growth Modeling and Analysis). In PAE, by taking the complex leaf overlap problem into consideration, the area of every plant is measured from top-view images in four steps. Given the abundant measurements obtained with PAE, in the second module GMA, a nonlinear growth model is applied to generate growth curves, followed by functional data analysis.

**Results:**

Experimental results on model plant *Arabidopsis thaliana *show that, compared to an existing approach, HPGA reduces the error rate of measuring plant area by half. The application of HPGA on the *cfq *mutant plants under fluctuating light reveals the correlation between low photosynthetic rates and small plant area (compared to wild type), which raises a hypothesis that knocking out cfq changes the sensitivity of the energy distribution under fluctuating light conditions to repress leaf growth.

**Availability:**

HPGA is available at http://www.msu.edu/~jinchen/HPGA.

## Introduction

**Figure 1:**
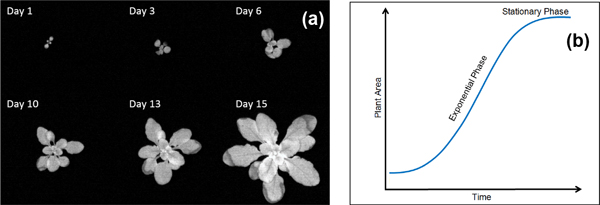
**An example of plant growth and an idealized general curve of typical higher plants**. (a) Top-view fluorescence images of *Arabidopsis *wild type growing over 15 days. (b) An idealized growth curve of typical higher plants.

Growth is the increase in dry mass, volume, length, or area that results from the division, expansion, and differentiation of cells [1] (Figure 1a). Plant growth is a fundamental biological process studied in a wide range of scientific fields, integrating across scales from physiology to community dynamics and ecosystem properties [2]. Computational plant growth modeling [3,4] enables a deeper understanding and more accurate predictions for a wide range of plant physiological problems. Specifically, in the studies of the key biological pathways responsive to bioitic or abotic stresses, probably the only way to capture the exact changes in plant growth rate (which may reflect how these pathways control photosynthesis activity or energy distribution) is to develop a precise and more efficient computational plant growth model.

In order to precisely model plant growth rate, we present a new computational model called HPGA (High-throughput Plant Growth Analysis) which identifies leaf tips and then uses the short curvature areas around them to estimate the area of each leaf individually, regardless whether they overlap or not. Then a nonlinear model is trained to learn growth characteristics in different development stages.

Plant growth models quantify two kinds of measurements: absolute growth rate (AGR) and relative growth rate (RGR), both of which require measuring biomass or plant area at successive time points. Traditionally, plant growth is often fitted with linear or exponential equations such as a logistic model (one- or two-parameter version) [5,6], comprising of initial exponential growth and a term that reduces RGR as the area increases, resulting in an asymptotic maximum area (Figure 1b) [7]. However, although simple logistic models require relatively few observations, they do not often fit with observations well [4]. In fact, there is an increasing amount of contemporary research suggesting that traditional approaches to modeling growth using linear and exponential models are inadequate due to their oversimplified assumptions [4]. Consequently, researchers have started to apply nonlinear models, including three- and four-parameter logistic [8,9], power-law, Gompertz [10,11] and monomolecular model [11,12] to provide enough flexibility to obtain the best fit between models and observations [4]. To achieve satisfactory results, all these models need abundant observations [4], which require either a labor-intensive protocol to frequently measure plant areas manually, or an automated phenotyping approach using computational measurements of plant area. Furthermore, in large-scale screen experiments where hundreds of plants are monitored simultaneously, manual inspection may not be an option. It is necessary to automate the plant area measurement and therefore recognize emergent growth phenotypes.

Taking advantage of the current rapid development in imaging systems and computer vision algorithms, high-throughput computational phenotyping techniques to non-invasively monitor plant growth have been developed [3,13-15]. In these approaches, top-view images are captured periodically and a growth curve is generated using the observed pixels of the plant area over time [3,16,17]. However, the observed pixels is remarkably affected by complex leaf overlap during growth (in addition to leaf twisting and curling, and circadian movement), resulting in inaccurate growth patterns. For old plants with many overlapping large leaves, the bias becomes more severe (Supplementary Fig S1). Mokhtarpour et al [18] have setup a three-camera system with two side views and one top view to correct for leaf overlapping areas, but the setup of side-view cameras is not suitable in many cases, e.g. large-scale screen of many plants simultaneously.

Since the observed value from a top-view will often cause problems in modeling plant growth, and since there is an emerging research demand for plant high-throughput phenotyping, more advanced approaches for plant growth analysis need to be developed. In this paper, a new computational model HPGA is presented to estimate leaf overlap percentage to measure plant area more precisely. Our approach has the following advantages.

• Unlike the existing approaches that simply counts the number of valid pixels in an image [3,13-15], HPGA estimates plant areas by explicitly taking leaf overlaps into consideration. Specifically, with a leaf development model [19], we address the leaf overlap problem with a four-step approach: plant center identification, leaf tip identification, leaf area estimation and plant area measurement.

• Our approach avoids the leaf segmentation problem to recognize all the leaves of a plant from a top-view image, which has been considered to be a challenging problem in the computer vision community due to high plant-to-plant variations (biodiversity) [20].

• With our high-throughput phenotyping technique, researchers are able to generate hundreds or even thousands of observations for every plant automatically. Feeding enough observations to a nonlinear model ensures the robustness and precision of plant growth modeling.

• In HPGA, functional data analysis is applied on growth curves for better interpretation of the plant growth scenarios. In our experiment, the coupling of photosynthetic and growth rate phenotypes raises an important hypothesis about gene function.

In summary, our study highlights a cost-effective, high-throughput phenotyping approach that, coupled with other phenotyping and genotyping techniques, facilitates the dissection of the dynamics of plant growth and development under varying environmental conditions.

## Methods

HPGA is composed of two independent modules, PAE (Plant Area Estimation) and GMA (Growth Modeling and Analysis). In PAE, the plant area is measured in four steps which will be described in the following text. Given the abundant measurements obtained with PAE, in the second module (GMA), a nonlinear growth model is applied to generate a growth curve for each plant, followed by functional data analysis. Modularity in design combines the advantages of standardization with those of customization [21]. In our case, it allows researchers, for example, to explore different nonlinear models without interfering the other parts of the algorithm.

### Plant area estimation

**Figure 2:**
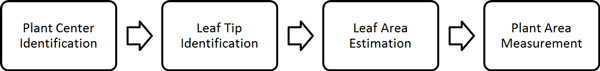
**Workflow of Plant Area Estimation (PAE)**.

The most straightforward approach to measure plant area which takes leaf overlap into account is to segment every leaf, measure leaf areas and sum them up. However, given the complex and diverse plant layout, the first step, leaf segmentation, is challenging [20]. Alternatively, we observe that although it is difficult to segment a complete leaf edge, it is practical to obtain a relative small curvature area near every leaf tip, which provides enough information about leaf shape, length and area, since there is a strong correlation among these features [19]. We propose PAE (Plant Area Estimation), a four-step approach to precisely measure the plant area even if leaves are heavily overlapped. The workflow of PAE is shown in Figure 2.

#### Plant center identification

The center of a plant is the geometric location where a plant starts to grow. In general, a plant center is the center of mass because of the symmetry of plants [22]. But in many other cases, due to the loss of leaves or light direction changes, a plant center is different from the geometric center. Therefore, instead of using the whole plant geometric properties, we develop a new method to identify plant center from a top-view image. The idea is to subdivide a top-view image into constituent regions, recognize a few leaves (≥ 2), and then locate the plant center according to leaf orientations, in that all the leaves arise from the center of a plant.

**Figure 3:**
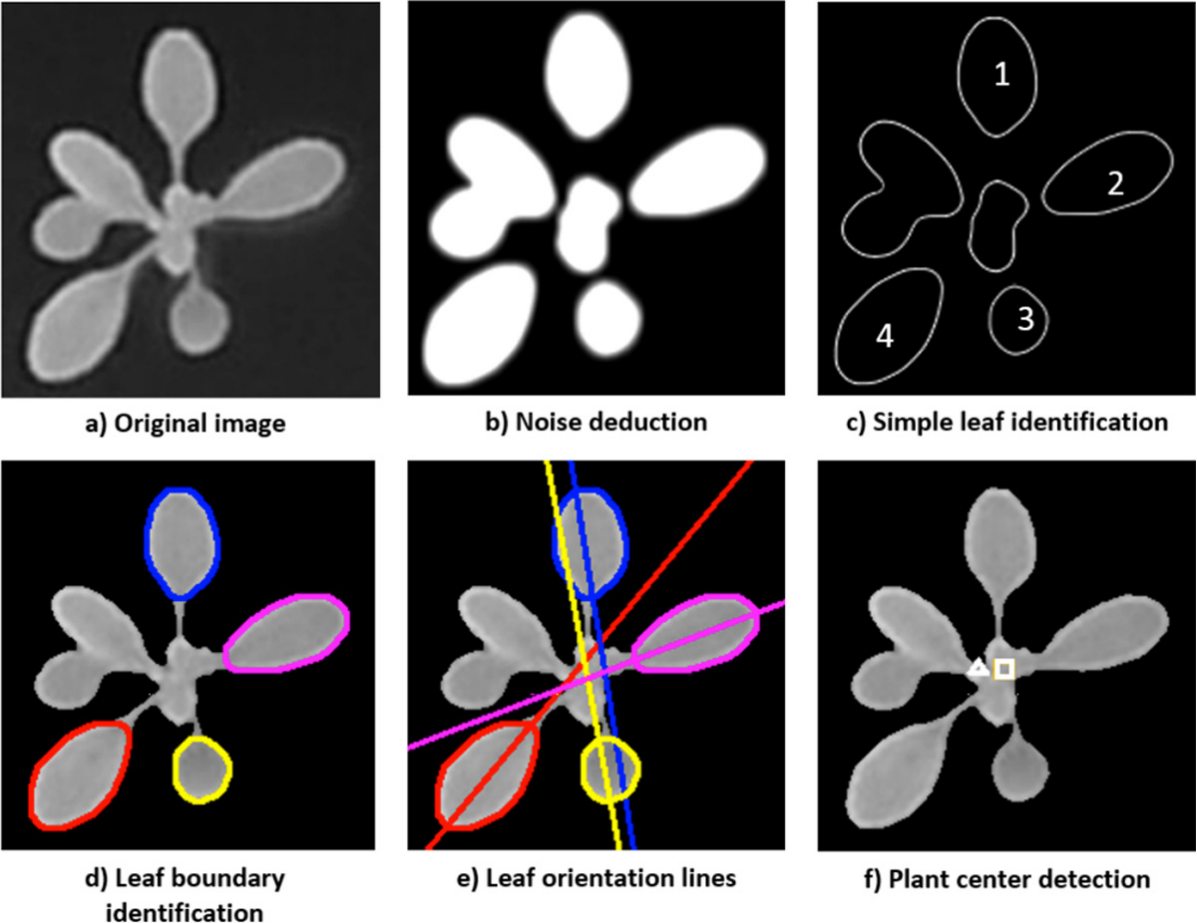
**PAE step 1: plant center identification**. The plant center found by using a few segmented simple leaves (square in (f)) is physiologically more reasonable than the center mass (triangle in (f)).

In the leaf segmentation step, the goal is to accurately identify a few leaves. There are two kinds of leaves in a top-view image: simple leaves and leaf complexes. Simple leaves refer to non-overlapping and separated leaves. On the contrary, leaf complexes are either young leaves that are fused together or overlapping large leaves. To avoid utilizing complicated leaf segmentation approach to identify all the leaves, which is often prone to errors, only the simple leaves are considered here. An example is shown in Figure 3 illustrating the idea of leaf segmentation, where Figure 3a is the original fluorescence image (12-bit gray scale) of a plant with four simple leaves and two leaf complexes. Here fluorescence images are used in order to observe the photosynthesis activity and the growth of the plants simultaneously.

We first apply Gaussian smoothing [23], a common approach for blurring images and removing details and noises, to reduce the noise level in the top-view images (Figure 3b). Then, Laplacian detector [23] is used to identify simple leaves. As a second-order derivative operator, Laplacian detector is suitable for edge location by looking for zero crossings. With it, the edges of simple leaves can be well-isolated, while the edges of leaf complexes are still crossed. Therefore, the regions containing only simple leaves are correctly detected (Figure 3c). Since the second-order derivative is extremely sensitive to noise, a fine edge detection approach called Canny edge detector [23,24] (the most commonly applied edge detector), is employed to accurately identify intensity discontinuities that define the leaf edges of all the simple leaves (Figure 3d).

After obtaining *n *segmented leaves (*n *≥ 2), we determine the leaf orientation for each leaf with a "leaf orientation line" which is the longest line across the leaf (Figure 3e). For a round leaf the petiole that connects to the leaf is used as additional information to determine the leaf orientation. Based on all the leaf orientation lines, a plant center is considered to be the point that is the closest to all the lines (the square in Figure 3f). Mathematically, given all the leaf orientation lines {*l*_1_, *l*_2_, ..., *l_n_*}, a plant center *c *is a point that:

(1) $$c=argmin_{c}\overset{n}{\underset{i=1}{\sum }}Dist\left(l_{i},c\right)$$

where *Dist*(*l_i_*, *c*) is the perpendicular distance from *c *to line *l*_*i*_. In summary, we developed a plant center identification method based on a few segmented simple leaves. It is theoretically more reasonable than to use the center of mass (the square against the triangle in Figure 3f).

#### Leaf tip identification

**Figure 4:**
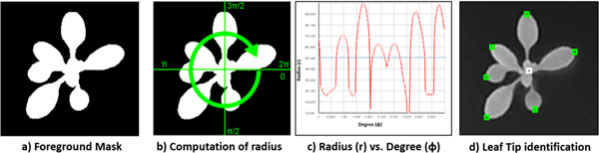
**PAE step 2: leaf tip identification**.

With the recognized plant centers, we identify all the leaf tips with three digital image process algorithms applied successively. First, a binary mask is created, distinguishing foreground (plant) from the background. This is done by dilating the output of the Sobel edge detector [23], which is a linear filter that computes the gradient by using a discrete differences between rows and columns of a 3-by-**3 **neighborhood, followed by the application of a flood fill algorithm [25] from the four corners of the image to determine the connected background area, similar to the "bucket fill" tool of paint programs. An example shown in Figure 4a is the binary foreground mask of the fluorescence image in Figure 3a.

Next, starting from a plant center *c*, a horizontal line is drawn to the right and rotated 360 degrees. For each degree *ϕ*, we compute the distance from the plant center to the outermost edge of the foreground mask (Figure 4b). This yields a vector of pairs of radius *r *and degree *ϕ *which describes, in polar coordinates, the shape of the outermost edge of the foreground mask(Figure 4c), with each peak representing a leaf tip.

**Figure 5:**
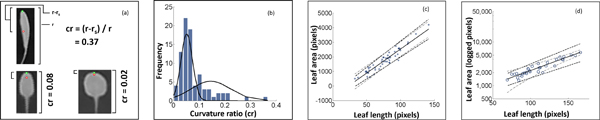
**PAE step 3: leaf area estimation**. In (c) and (d), each point represents a leaf, the 95% confidence intervals are the dashed lines, and the 95% prediction intervals are the grey dotted dash lines. (a) The definition of "curvature ratio" (*cr*). (b) The distribution of *cr *in the training data. (c) The linear relationship between leaf length and leaf area if *cr <*0.07. (d) The exponential relationship between leaf length and leaf area if *cr *≥ 0.07.

In the third step, to avoid repeatedly counting the same leaf tips, we smooth the radius-degree vector by applying a moving average operation [26] that calculates the unweighed mean of the radius in a window. Mathematically, given a degree *ϕ*, the smoothed radius *r_s_*(*ϕ*) is:

(2) $$r_{s}\left(\phi \right)\;=\;\frac{\sum_{i=1}^{k}Radius\left(\phi ,i\right)}{k}$$

where *Radius*(*ϕ*, *i*) is the *ith *radius flanking *ϕ*, and *k *is the window size. An example of the smoothed radius for three types of leaves is shown in Figure 5a. However, we do not directly consider every local maximum in the smoothed radius vector to be a separate leaf tip, because the radius smoothing operation always underestimates the leaf radius. Alternatively, we consider the non-smoothed radius *r *at every *ϕ_max_local_*, the corresponding degree of the local maximum in the smoothed radius vector, to be a separate leaf tip (denoted as *t*). A radius threshold *T_r _*(the dotted line in Figure 4c) is applied to avoid capturing false leaf tips when radius is very small.

Our approach ensures the successful identification of all the outer leaf tips, as shown in Figure 4d. Its performance may be reduced if a plant is over-grown which, in fact, rarely happens in leaf based scientific experiments.

#### Leaf area estimation

In early leaf development, because of cell proliferation, the primordium (formed by apical and marginal meristems) undergoes a *slow *limited expansion phase, followed by a *rapid *dramatic expansion phase, principally because of cell expansion [19]. Starting with this synthetic model, recent studies reveal that there is a strong relationship between leaf area and leaf properties such as primary and secondary vein density [18,19]. In Sack *et al*., a log-linear relationship between leaf area and leaf dimension (length and width) has been discovered with a significant p-value [19]. However, in our situation it is difficult to measure the leaf width because of the challenging leaf segmentation problem. Therefore, we develop a new leaf length-to-area model to infer the leaf area with leaf length and a small area around leaf tip.

We first define the leaf length as the distance from the base of the petiole of the leaf to the outermost point on the leaf. In our model, we assume that all the leaves have petiole bases at the same point, *i.e*., the center of the plant. Therefore, a leaf length is estimated as being the distance from the plant center to the leaf tip, which is the radius (*r*). We include the petiole length because of two reasons. First, it is difficult to identify the leaf bottom because of the leaf overlap problem. Second, the leaf length and petiole length are usually proportional, except for a few genetic or natural variations. Consequently, the proportion can be modeled in the leaf area inference.

Due to the diversity of leaf shapes, leaves with the same length can have very different areas. To this end, we define "curvature ratio" *cr *= (*r *- *r_s_*)*/r*, where *r *is the leaf length and *r_s _*is the smoothed leaf length defined in Eq 2, to describe the shape of the small area around leaf tip (Figure 5a). Curvature ratio is an indicator of leaf shape, because the leaf edge is usually smooth, the small area around leaf tip, which is the easiest to obtain, has the complete information of leaf shape.

Next, we learn the relationship between leaf area and the features we collected, *i.e*., the leaf length r and the curvature ratio *cr*. Similar to the approach in Sack *et al *[19], 87 leaves of wild type (WT) *Arabidopsis *plants were collected with the plant ages ranging from 1-5 weeks old, and then their leaf areas, lengths and curvature ratios were attributed. In our training data, the distribution of *cr *fits a mixed Gaussian distribution (Figure 5b). Based on this, a cutoff of *cr *at 0.07 was selected to split all the leaves into two subsets: the round leaves and the elongated leaves. For each of the leaf subset, the relationship between leaf area and leaf length is fit to the best linear or exponential model (Eq 3). Leaves below the cutoff shows a linear relation between length and area (Figure 5c) with coefficient of determination (*R*^2^) value 0.90, while leaves above the cutoff exhibited an exponential relationship (Figure 5d) with *R^2 ^*value 0.88. By dividing the leaf length-to-area model into two categories, we are able to increase the coefficient of determination (*R*^2^) from 0.87 to 0.90 (*cr *< 0.07) and 0.88 (*cr *≥ 0.07) respectively.

(3) $$l=\left\{\begin{array}{c} \text{32}.\text{9 }\;\times \;r-\text{769}.\text{9 }\;\;\text{if }cr<0.0\text{7}\\ \text{513}.\text{8 }\;\times \;e^{0.0\text{146 }\times \text{ r}}\;\;\;\text{otherwise} \end{array}\right)\;$$

where *I *is the leaf area, *cr *is the leaf curvature ratio and *r *is the leaf length. The 95% confidence intervals (CI) for *cr *< 0.07 are (29.9, 36.0) and (-1012.0, -527.6); 95% CI for *cr *≥ 0.07 are (406.1, 621.6) and (0.0130, 0.0162), meaning 24.8% and 39.7% maximal leaf-to-leaf variances respectively.

#### Plant area measurement

The summary of all the leaf areas is an important component of the whole plant area. But it should also be noted that some inner leaf tips may sometimes be missed due to the leaf tip threshold *T_r _*or smoothing window width *k*. Therefore, adjustments should be applied in the plant area measurement by taking both the observed area from a top-view and the summary of the leaf areas into consideration. In this paper, plant area, denoted as *a*, is defined as:

(4) $$a=a_{l}\;.\;\left(\text{1}+p_{overlap}\right)=\;a_{l}\;.\;\left(1+\;\frac{abs\left(a_{t}-a_{l}\right)}{max\left(a_{t},a_{l}\right)}\right)$$

where *a_l _*is the summarized leaf area defined as $a_{l}=\;\sum_{i=1}^{m}l_{i}$(*l_i _*is the area of the *i*th identified leaf and *m *is the number of leaf tips of a plant), *a_t _*is the observed value of plant area from a top-view, and *p_overlap _*is the leaf overlap percentage which equals the absolute difference between *a_t _*and *a_l _*divided by the maximum value of the two. In Eq 4, if *p_overlap _= *0, plant area is exactly the summary of all the leaf areas whose tips are identified; otherwise, plant area is estimated with $a_{l}$ and $a_{t}$: if $a_{l}>a_{t},a=2\cdot a_{l}-a_{t}$, else $a=2\cdot a_{l\;}-\;a_{l}^{2}/a_{t}$.

### Growth modeling and analysis

Given the abundant plant area measures obtained with PAE, a nonlinear model is applied to generate precise growth curves which are suitable for the subsequent functional data analysis.

#### Nonlinear growth model

Among the basic functional forms for plant growth modelling, logistic model is the most commonly utilized asymptotic form [6,7,11]. It has one-, two-, three-, four- and five-parameter versions, where each version uses a logistic function to relate examinee ability and the parameter(s) to the growth responding to time [27]. The simple logistic models (one- and two-parameter versions, called 1PLM and 2PLM) do not often fit with observations well [4], although they require relatively few observations for the training.

In the three-parameter logistic model (3PLM), by relaxing the 2PLM requirements of the model to allow for a nonzero lower asymptote, the lower horizontal asymptote is set at *A*_0 _(the initial plant area), and the inflection point (the time at which AGR is maximized) falls rigidly at the time when the plant area is half of the upper horizontal asymptotes. It collapses to the exponential in the limit as the upper horizontal asymptotes approaches infinity. The model, as a function of time *t*, is:

(5) $$A\left(t\right)\;=\;\frac{A_{0}\cdot A_{a}}{A_{0}+\left(A_{a}-A_{0}\right)e^{-\gamma \cdot t}}$$

where *t *is time, *A*(*t*) is plant area at time *t *(modelled value, different from the plant area observation (a)), *A*_0 _is the initial plant area, *A_a _*indicates the upper horizontal asymptotes, and *γ *is an acceleration or deceleration parameter related to time. If *t = *0, *A = A*_0_*A_a _/*(*A*_0 _*+ *(*A_a _- A*_0_)) = *A*_0_; if *t *→ ∞, *A = A*_0_*A_a _/*(*A*_0 _*+ *(*A_a _- A*_0_)·0) = *A_a_*; if *A = *(*A_a _- A*_0_)*/*2, *A*(*t*)*" = *0.

The four-parameter logistic model (4PLM) looses one or the other of the constraints in 3PLM [8,28]. For some data, the additional flexibility of the four-parameter version greatly increases the variance explained by the model, although 3PLM provides a more parsimonious and equally adequate fit in other situations. The most general form of this is the five-parameter logistic model (5PLM) [9], which provides maximum flexibility and alleviates both restrictions.

Note that inappropriate functional forms will often fail to converge; or in other cases, the wrong form can result in convergence with unreasonable parameter estimates [4]. Therefore, we choose 3PLM for plant growth modeling to avoid over-parameterization, and use nonlinear least squares to fit the plant area observations to 3PLM.

#### Functional data analysis

Plants are self-assembled systems for solar harvesting. In the early stages of plant growth, harvested energy is used primarily for the creation of new light capture facilities (leaves), resulting in an exponential growth rate. As a plant matures, an increasing percentage of the energy captured can be redirected to storage for later harvest. Studying plant growth over time is essential towards the understanding of how plants manage resources at different ages or under different environmental stresses.

The absolute growth rate (AGR) and relative growth rate (RGR) by the plant area can be calculated as:

(6) $$AGR=\frac{A_{t}-A_{t-\Delta t}}{\Delta t}$$

(7) $$RGR=\frac{ln\left(A_{t}\right)-ln\left(A_{t-\Delta t}\right)}{\Delta t}$$

where *t *is time, Δ*t *is time interval and *A_x _*is the plant area at time *x*. Growth rate by mass can be further assessed using additional measures {*e.g*., leaf mass fraction (LMF) and unit leaf assimilation ratio (ULR)). Knowledge of AGR and RGR is critical for researches to relate growth to biomass, biofuels, and bioenergy.

(8) $$velocity=\frac{dA\left(t\right)}{dt}=\frac{A_{0}\cdot A_{a}\cdot \left(A_{a}-A_{0}\right)\cdot \gamma \cdot {\text{e}}^{-\gamma \cdot t}}{{\left(\left(A_{a}-A_{0}\right)\cdot e^{-\gamma \cdot t}+A_{0}\right)}^{2}}$$

(9) $$acceleration=\frac{dA^{2}\left(t\right)}{dt^{2}}=\frac{2A_{0}\cdot A_{a}\cdot {\left(A_{a}-A_{0}\right)}^{2}\cdot \gamma \cdot^{2}\cdot {\text{e}}^{-2\gamma \cdot x}}{{\left(\left(A_{a}-A_{0}\right)\cdot {\text{e}}^{-\gamma \cdot x}+A_{0}\right)}^{3}}-\frac{A_{0}\cdot A_{a}\cdot \left(A_{a}-A_{0}\right)\cdot \gamma^{2}\cdot {\text{e}}^{-\gamma \cdot x}}{{\left(\left(A_{a}-A_{0}\right)\cdot {\text{e}}^{-\gamma \cdot x}+A_{0}\right)}^{2}}$$

The rate of change of the plant area sometimes is more interesting than its actual value. To this end, we need to study what alters velocity (the first order derivative of the growth curve, Eq 8) and acceleration (the second order derivative of the growth curve, Eq 9) which is instantaneous curvature in a growth curve [29]. The smoothed growth curve generated with 3PLM is capable of giving a qualified impression of the velocity and acceleration of the plant growth.

## Experiments

**Figure 6:**
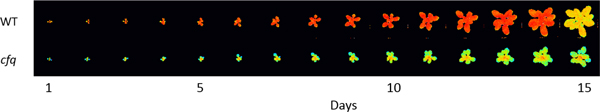
**Overview of the photosynthesis activity Φ*_II _*and growth of *cfq *and wild type (col-0) plants under fluctuating light conditions**. The mutant is clearly distinguished by its distinct area and colors (red and blue representing high and low Φ*_II _*values respectively).

To systematically evaluate HPGA and compare its performance with the existing approach for plant area estimation, a well-calibrated growth experiment was carried out. In the experiment, three wild type (col-0) plants and three *cfq *(AT3G24530; **c**oupling **f**actor **q**uick recovery) mutant *Arabidopsis thaliana *plants were grown side by side in a fluctuate light condition (Supplementary Fig S3) for 15 days from 10 days old from seedling to 25 days old, during which period rosette leaves grow the most rapidly [30]. A top-view fluorescence image was taken every 15 minutes during the day time, in order to observe the photosynthesis activity and the growth of the plants simultaneously for the dissection of general basis of plant growth and development under fluctuating light conditions. The overview of the experimental results is shown in Figure 6. In total, 960 fluorescence images were collected, preprocessed and fed to HPGA. We selected a small-scale experiment because we can manually identify the inferior growth of the mutant line and use them as the ground truth to evaluate the algorithm performance. Certainly, HPGA is designed for large-scale screen experiments with hundreds of plants monitored simultaneously.

cfq is an AAA-type ATPase family protein which is involved in ATP synthase regulation [31,32]. It harbors a mutation on the gamma subunit of the ATP synthase, which accelerates ATP synthetic activity at the cost of accumulating *pmf *(and consequently the Δ*pH *required for photoprotective, *q_E_*). Equilibrium redox titration revealed that this mutation makes the regulatory sulfhydryl group energetically much more difficult to reduce relative to the wild type [33]. The growth of the mutant, however, is not significantly impaired under standard laboratory growth conditions (constant light at 150 *μmol m*^-2 ^s^-1^) [33]. Here we investigate how the mutant affects growth under non-static light conditions, in that overlapping regulatory mechanisms can compensate for loss of some processes under artificially static lab conditions, but each underlying process may have different dynamic responses and may be activated under different sets of environmental conditions. In the following text, we first evaluate HPGA in terms of measuring plant area, followed by the dissection of the different growth patterns of the *cfq *and wild type plants.

### Results of plant area estimation

**Figure 7:**
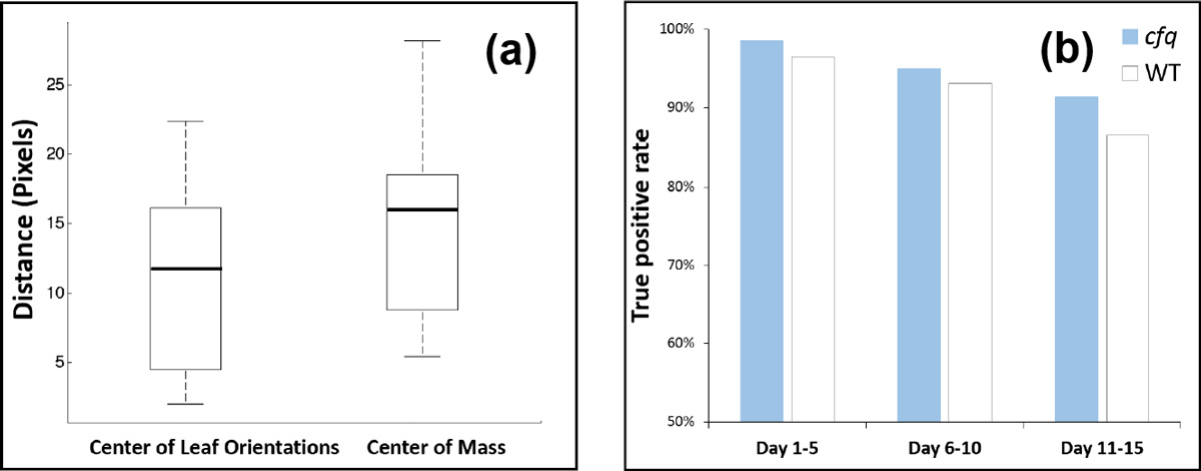
**Results on plant center identification and leaf tip identification**. (a) Plant center identification. (b) Leaf tip identification.

We first tested whether the plant centers are correctly localized with our leaf orientation based approach by randomly choosing 12 plant images and manually determining their actual centers. The shorter the distance from an actual center to the output of our algorithm, the better the algorithm is. In this analysis, we compared HPGA with the center of mass approach. The results shown in Figure 7a reveals that our leaf orientation based method is better (with shorter distance to actual plant center) than the center of mass approach (p-value 0.18 with a two-tailed t-test with unequal variance [34]). The p-value is insignificant because of the symmetry of plants [22]. Nevertheless, our approach provides practically more precise plant centers. In a growth experiment, a plant center does not move so the center of each plant was determined using a single image, which avoids applying the algorithm on plants that too small or too large.

The leaf tip identification algorithm was tested for all the images. The success rate of these identifications was 93.5% (on average). The true positive rate reduces gradually with increasing plant area (from 98.5% to 86.5%; Figure 7b, Table 1) due to the more complex plant layout. The true positive rate can be further elevated by tracking leaves in adjacent images to incrementally update leaf tips. This analysis also shows that the success rate of tip identification on the *cfq *plants is constantly higher than on the wild type plants by 2-5%, because the wild type plants have a more complex layout and more inner leaves (Figure 10b).

**Table 1:** Results on leaf tip identification (true positive rate).

Days	*cfq*				WT			
	
	Sample 1	Sample 2	Sample 3	Avg (*cfq*)	Sample 1	Sample 2	Sample 3	Avg(WT)
Day 1-5	100%	97%	99%	99%	98%	97%	94%	97%

Day 6-10	97%	96%	92%	95%	97%	89%	94%	93%

Day 11-15	94%	88%	92%	91%	89%	89%	82%	86%

**Figure 8:**
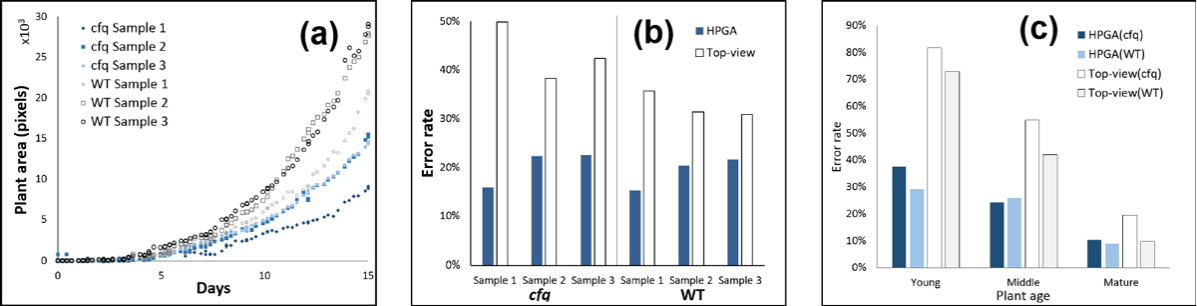
**Results on plant area measurement**. (a) Results of plant area measurement with HPGA. (b) Comparison of HPGA with the observed value from a top-view by plants. (c) Comparison of HPGA with the observed value from a top-view by age.

**Figure 9:**
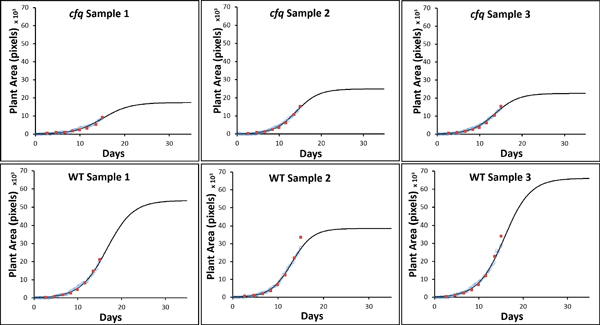
**Growth curves (black) for six plants generated using 3PLM**. The blue circles on day 1-15 represent plant areas measured with HPGA and the red squares are the actual plant areas.

Finally, the plant areas were computed using HPGA on all of the images (Figure 8a). For the purpose of evaluation, eight plant areas were manually measured for each of the six plants (young, middle and mature, in total 48 plant areas), and compared to HPGA with the observed value of plant area from a top-view (the existing common approach for plant area estimation [3], which ignores the leaf overlap problem). In the manual plant area measurement process, every leaf on an image was manually segmented by a researcher and its edge was completed if it is partially covered by other leaves. The unit for all the measurements is the number of pixels. The comparison results in Figure 8b shows that HPGA is consistently better than directly using the observed value of plant area from a top-view, with half of the error rate (19.7% against 38.1%). Specifically, it reduces the error rate from 43.5% to 20.3% for the *cfq *plants and reduces the error rate from 32.7% to 19.1% for the wild type plants. In addition, Figure 8c shows that HPGA is more accurate for young and middle age plants, and its performance is similar to the top-view observations when plants are mature. Figure 9 shows how the results of HPGA match the actual (manually measured) values. Overall, the analysis suggests that HPGA is a reliable high-throughput plant area measuring platform for both wild type and the mutant from young to mature plants.

**Figure 10:**
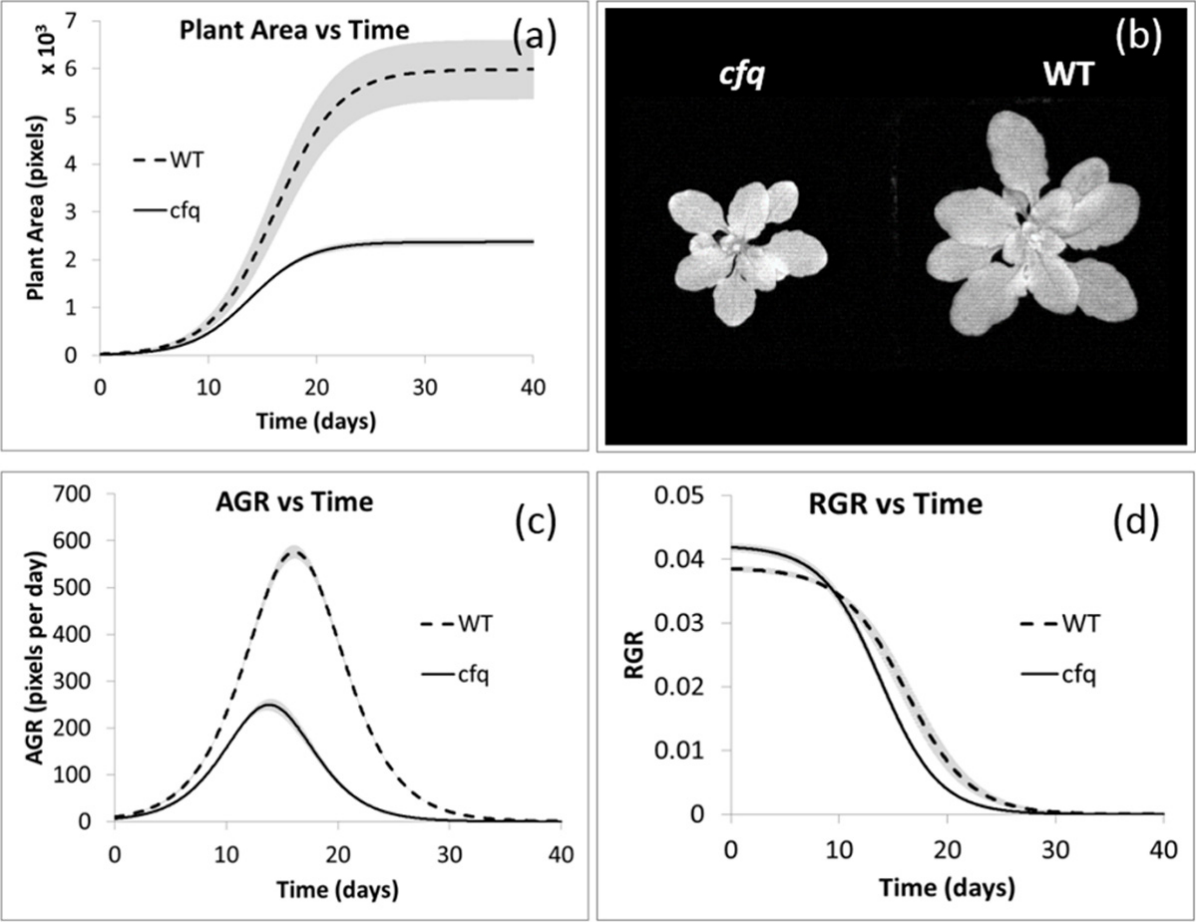
**Growth analysis of *cfq *and wild type plants**. (a) averaged growth curve of *cfq *and wild type. (b) *cfq *and wild type plants on the 15th day of the experiment, which is 25 days from seedling. (c) absolute growth rate. (d) relative growth rate. The solid and dashed lines in (a), (c) and (d) represent the averaged growth curves of *cfq *and wild type plants respectively; and the grey areas represent the one standard deviation of uncertainty.

### Results on growth modeling and analysis

Using all of the output data from HPGA as observations, growth curves were generated for each of the six plants using a three-parameter logistic model (3PLM) (Eq 5). The solid line in Figure 9 is the growth curve, the circles represent the plant areas obtained with HPGA, and the squares are the actual plant area measured manually. The learned parameters of 3PLM and their 95% CI are listed in Table 2. It shows that the 3PLM growth curves correlate well with the plant areas.

**Table 2:** Parameters of 3PLM and their upper and lower bounds with 95% confidence.

Plant	Parameters			Lower bound of 95% Cl			Upper bound of 95% Cl		
	
	*γ*	*A*_0_	*A*_*a*_	*γ*	*A*_0_	*A_a_*	*γ*	*A*_0_	*A_a_*
*cfq *Sample 1	0.03711	129.9	17440	0.0334	93.55	12780	0.04081	166.3	22100

*cfq *Sample 2	0.04296	127.2	24850	0.03984	95.37	21300	0.04607	159.1	28390

*cfq *Sample 3	0.04128	167.3	22590	0.03916	139.9	20540	0.04340	194.7	24650

WT Sample 1	0.03823	210.1	53640	0.03502	156.2	35500	0.04144	263.9	71770

WT Sample 2	0.05020	127.5	38570	0.04835	107.7	36640	0.05205	147.3	40500

WT Sample 3	0.03907	304.8	66060	0.03656	243.8	52480	0.04159	365.9	79640

*A*_0 _is the initial plant area, *A*_*a *_is the upper horizontal asymptotes, *γ *is an acceleration or deceleration parameter related to time.

The growth model reveals two interesting results. First, unlike the growth pattern under normal static light conditions, which is similar for wild type and *cfq *plants [33], the upper horizontal asymptotes of the wild type plants (~65, 000 pixels) is almost three times that of the *cfq *plants (~24, 000 pixels) (Table 2), suggesting that by knocking out the cfq gene, plants have much less potential to grow under fluctuating light conditions. The slower growth rate of the mutant correlates well with diminished photosynthetic efficiency compared to the wild type (Figure 6), consistent with the higher energy requirement for sustained activation of the ATP synthase and a lower capacity of ATP synthesis in the mutant [33]. The efficiency of *cfq *is continuously repressed or unable to recover under fluctuating light. This is probably because of the significantly lower light-induced ATPase and ATP synthase activity in the mutant compared with the wild type [33]. In summary, the low photosynthetic rates correlates well with the small plant area of the *cfq *plants, raising a hypothesis that knocking out the cfq gene changes the sensitivity of the energy distribution under fluctuating light conditions to repress leaf growth.

Second, in the growth curve, the wild type plants reach their upper horizontal asymptotes at around 25 days from the start of our experiment, which is 35 days from seedling. This matches with the *Arabidopsis *growth stage description, which states that the rosette growth of wild type plants completes after 29.3 days from seedling with a standard deviation of 3.5 days [30]. The *cfq *plants reach the upper horizontal asymptotes at around 20 days from the start of our experiment. This shorter growth period (5 days less), combined with the decreased growth rate, results in a smaller rosette leaf surface area in *cfq*. The values of the *cfq *sample 1 and wild type sample 2 are statistically different from the rests (p-value 7.1E-14 and 3.6E-09 respectively with a two-tailed t-test with unequal variance [34]; Supplementary Fig S2), probably because of natural biological variability. Therefore, they were excluded in the downstream growth analysis.

The AGR and RGR of *cfq *and wild type plants were calculated using Eq 6 and *7 *(respectively) with Δ*t = *0.35 days. The averaged results shown in Figure 10a, b reveal distinctive growth patterns between the two kinds of plants. The wild type plant is almost three times of *cfq *when reaching the upper horizontal asymptote, and it have significantly higher AGR than *cfq *(Figure 10c). The peak of AGR of the wild type plants is 2.28 days later than the peak of *cfq*. The RGR of *cfq *is slight higher (0.003) than that of the wild type plants during the first a few days. The decreasing rates of RGR of both kinds of plants are very similar, which means the RGR of the *cfq *plants was shifted for about 2.5 days to the left, limiting the plant to grow at a fast rate for a shorter period of time (Figure 10d).

## Discussion

The experiments on *Arabidopsis thaliana *wild type and *cfq *mutant plants show that HPGA reduces the error rate of measuring plant area by half in average if compared with the existing approaches. The low photosynthetic rates and small plant area of *cfq *suggests that knocking out cfq changes the sensitivity of the energy distribution under fluctuating light conditions to repress leaf growth. If it is true, a key question regarding to growth is whether the plant size difference changes linearly all through the developmental stages, or it actually varies and there is a dominant period. To answer this question, the following functional analysis on the growth curves was conducted.

**Figure 11:**
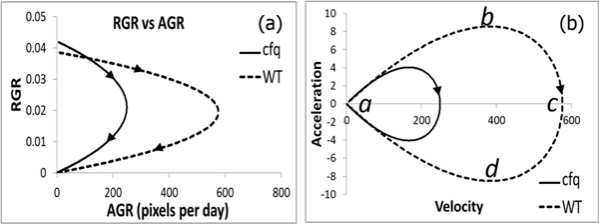
**Functional analysis of *cfq *and wild type plants**. (a) relative growth rate against absolute growth rate. (b) phase-plane plot of velocity against acceleration.

First, we generated the curves in the AGR against RGR plot (Figure 11a), in which the x-axis is the AGR and y-axis is RGR. It shows that there is a cross of *cfq *and wild type curves in the early period. Before the crossing the AGR of the *cfq *plants is lower than that of the wild type plants while its RGR is higher than the latter. It suggests that the area of the young *cfq *plants (before the measurement starts) is smaller than the wild type plants, which consists with an earlier discovery that the total chlorophyll content in *cfq *grown at low light is lower than that in the wild-type plants [33].

Second, in Figure 11b, a phase-plane plot of acceleration against velocity describes a basic harmonic process that bounces between two states: potential and kinetic [29]. In terms of plant growth, potential corresponds to resources that are available to bring about some growth activity such as cell proliferation, and kinetic corresponds to the process when the resources are consuming (e.g. cell expansion). At the point that a plant starts to grow from its lower horizontal asymptote, its potential and kinetic are both zero (Figure 11b point *a*)*; *it overlaps with the point that a plant reaches its upper horizontal asymptote. Potential is also zero when kinetic is maximized (Figure 11b point *c*) which happens when plant area increase is half of its maximum value ((*A_a _- A*_0_)*/2*), *i.e*., *A*(*t*)*" = *0. In the same figure, point *b *means no kinetic but maximal potential, and point *d *means no kinetic but maximal negative potential. The two points represent two critical time points of growth, probably related to the turn-over points of cell proliferation, suggesting that point *b *relates to the most active cell proliferation and point *d *relates to the most inhibition to cell proliferation.

In our experiments, the wild type plants reach point *b *at acceleration 8.6 and velocity 384.7 on the 12th day and reach point *d *at acceleration -8.5 and velocity -389.1 on the 20th day, with the absolute acceleration values more than twice as high as that of *cfq *(4.0 on the 10th day and -4.1 on the 17th day), suggesting the regulation of cell proliferation in *cfq *is much less active than wild type under fluctuating light. The period from point *b *to *d *is usually defined as the fast growing period. While the wild type plants spent 8 days in the period, *cfq *plants only spent 7 days and have much lower acceleration and velocity, resulting in much smaller plant areas. The covered area in the phase-plane plot is proportional to the amount of energy transferred during the process. The area ratio 4.88 (WT 6819 against *cfq *1398) indicates that in *cfq *much less energy has been distributed to the growth.

## Conclusion

HPGA is a high-throughput phenotyping platform for plant growth modeling and functional analysis. It has two components, PAE (Plant Area Estimation) and GMA (Growth Modeling and Analysis). In PAE, by taking the complex leaf overlap problem into consideration, the area of every plant is measured from top-view images in four steps. In GMA, a nonlinear growth model is applied to generate growth curves, followed by functional data analysis. HPGA addresses the leaf overlap problem by counting leaves and then measuring leaf lengths. It avoids segmenting all the leaves from a top-view image, which is extremely challenging [20]. Feeding enough and high-quality plant area measures to a nonlinear model (3PLM) ensures the robustness and precision of nonlinear plant growth estimation.

The major contribution in HPGA is a new plant area measurement which takes leaf overlap into consideration. We also noticed that the common approach in fluorescence image segmentation is global thresholding [16-18,35]. However, in a dynamic/natural condition, fluorescence intensity varies from time to time, so that applying a fixed global threshold for all the images may result in significant artifacts and consequently not practical for our study. In HPGA, we processed the images with a reasonable assemble of current images processing techniques that are clearly better than the global thresholding approach.

In HPGA, the leaf length-to-area model is genome specific. Changing from one species to another needs to train the model again with the new leaves. Seeking new ways to relate leaf area to detectable attributes is an essential future work. HPGA is developed for plant science research focusing on 2D plant *Arabidopsis thaliana*. Without knowing the height of each leaf, it is impossible to apply HPGA on any 3D plants that are seen more often in the field. By adding more cameras, we plan to extend HPGA to model the growth of 3D plants such as camelina, tobacco, tomato and bean. Furthermore, it is frequently desirable to develop a growth model with biologically interpretable parameters, which shall be addressed in our future model as well.

## Authors' contributions

DMK and JC conceived the project. JAC conducted the *Arabidopsis *growth and photosynthesis experiment. TLO and YJ designed and implemented the algorithm, built the web site, and finished the computational experiment. All prepared the manuscript.

## Competing interests

The authors declare that they have no competing interests.

## Declarations

The funding to support the publication fees is Chemical Sciences, Geosciences and Biosciences Division, Office of Basic Energy Sciences, Office of Science, U.S. Department of Energy (grant no. DE-FG02-91ER20021) to DMK and JC.

This article has been published as part of *BMC Systems Biology *Volume 7 Supplement 6, 2013: Selected articles from the 24th International Conference on Genome Informatics (GIW2013). The full contents of the supplement are available online at http://www.biomedcentral.com/bmcsystbiol/supplements/7/S6.
